# Sleep Patterns, Eating Behavior and the Risk of Noncommunicable Diseases

**DOI:** 10.3390/nu15112462

**Published:** 2023-05-25

**Authors:** Sofia Gomes, Cátia Ramalhete, Isabel Ferreira, Manuel Bicho, Ana Valente

**Affiliations:** 1 ATLÂNTICA-University Institute, 2730-036 Barcarena, Portugal; 2 Research Institute for Medicines (iMed.ULisboa), Faculty of Pharmacy, Universidade de Lisboa, Av. Prof. Gama Pinto, 1649-003 Lisbon, Portugal; 3 Ecogenetics and Human Health Research Group, Environmental Health Institute (ISAMB), Associate Laboratory TERRA, Faculty of Medicine (FMUL), University of Lisbon, 1649-028 Lisbon, Portugal; 4 Instituto de Investigação Científica Bento da Rocha Cabral, Calçada Bento da Rocha Cabral 14, 1250-012 Lisbon, Portugal

**Keywords:** sleep patterns, metabolism, hormones, appetite, chronic diseases

## Abstract

Sleep is extremely important for the homeostasis of the organism. In recent years, various studies have been carried out to address factors related to sleep patterns and their influence on food choices, as well as on the onset of chronic noncommunicable diseases. The aim of this article is to provide a scientific literature review on the possible role of sleep patterns on eating behavior and the risk of noncommunicable diseases. A search was performed on Medline (PubMed interface) using several keywords (e.g., “Factors Influencing Sleep” OR “Sleep and Chronic Diseases”). Articles published between 2000 and the present date that relate sleep to cyclic metabolic processes and changes in eating behavior were selected. Changes in sleep patterns are increasingly detected today, and these modifications are mainly caused by work and lifestyle conditions as well as a growing dependence on electronic devices. Sleep deprivation and the resultant short sleep duration lead to an increased appetite via an increase in the hunger hormone (ghrelin) and a decrease in the satiety hormone (leptin). Nowadays, sleep is undervalued, and thus often impaired, with consequences for the performance of various body systems. Sleep deprivation alters physiological homeostasis and influences eating behavior as well as the onset of chronic diseases.

## 1. Introduction

Sleep is of extreme importance for the adequate performance of the organism, not only accounting for inactive periods [1] but as a daily process of physiological recovery [2], and it should not be considered optional [1] due to its recognized health benefits [3,4]. Sleep is important for cognitive and motor functions [1] and is a critical gateway for energy repositioning and infection control [3]. Sleep is a fundamental biological need and its absence and/or reduction is recognized to have several consequences on the metabolism and cognitive function of human beings [5]. Since the industrial era, the circadian rhythm of sleep–wake habits has changed [3]; in modern society, also known as a 24 h society [6], it is quite common to shorten sleep [2,7,8] and/or to be chronically deprived of it [2,9,10] due to many activities carried out at night, namely leisure activities [9,11], or night shift work [12]. Sleep is an important daily process of physiological recovery, but in modern society, sleep duration is increasingly shorter, and this may have harmful effects on endocrine, immunological [2,13,14], cardiovascular [13,14], neurological [14] and cognitive systems, and may thus lead to the development of chronic diseases [2]. Shortened sleep also causes a decrease in energy expenditure, since sleeping fewer hours leads to the early appearance of fatigue and to a decrease in physical activity [15,16]. The quality of sleep due to high technological development in modern societies has a very negative impact on sleep efficiency [7]. Thus, individuals with shortened sleep patterns are at greater risk of deteriorating health, affecting metabolism [10,17] and consequent onset of chronic diseases [15,18,19]. In recent years, studies have been carried out with the aim of analyzing factors that influence sleep, the consequences of distinct sleep patterns on metabolism and their influence on food choices, which can lead to the development of several major chronic diseases. The present work aims to carry out a scientific literature review focusing on the possible effects of sleep patterns on eating behavior and the risk of noncommunicable diseases, with a focus on children, adolescents, and healthy adults.

## 2. Materials and Methods

This scientific literature review was carried out on scientific articles and the search engine used was the PubMed database. A combination of several keywords was used, such as Chronotype, Sleep Patterns, Factors that influence Sleep, Sleep and Metabolism, Sleep and Chronic Diseases, Sleep and Obesity, Sleep and Diabetes, Sleep and Cardiovascular Diseases, Sleep and Depression, and Sleep and Sleep Apnea. Without applying any inclusion criteria, the search resulted in 95,252 articles. After applying the inclusion criteria—studies carried out between 2000 and 2022 in humans, presenting both in the title and in the abstract the keywords “sleep” and “Chronic noncommunicable diseases” and being fully available for reading—the total number of articles available for this review was 2280. After reading the abstracts of the various articles, those that were considered important were selected, totaling 112 articles.

## 3. Results

### 3.1. Factors That Affect Sleep

For humans, who are a diurnal species, after a regular day in which they wake up and are active for a long period of time, there is a feeling of nocturnal tiredness that ends with a night of rest [20]. This sleep–wake cycle is due to the interaction between homeostatic and circadian processes [21]. A good example of this interaction is the external environmental light–dark signals that, when constantly transmitted to the organism, lead to the activation of clock genes [22]. These genes, found in the suprachiasmatic nucleus of the hypothalamus and in peripheral tissues, are responsible for modulating the behavioral and biological circadian rhythms [23] in anticipation of changes in routines and adjusting behavior [24]. The urge to sleep can occur at any time of the day, as it happens with individuals who engage in night shift activities [20].

However, all individuals have a unique circadian sleep–wake preference, which can be called a chronotype [25,26,27]. There are three known chronotypes: morningness, eveningness and midrange/intermediate [25,26,27,28,29,30,31]. Individuals with the morningness chronotype go to sleep and wake up early, reaching peak physical and mental performance, body temperature, cortisol, and melatonin at the beginning of the day [29,31]. People with this type of chronotype have regular sleep patterns over working days and rest days [31]. By contrast, people with the eveningness chronotype go to sleep and wake up late, experiencing social jet lag, that is, a discrepancy between their biological and social times, resulting in a better performance at the end of the day, feeling more tired and reporting poor sleep quality [29,31]. Circadian preferences are determined from an early age, by both biological and social factors [30], but there are studies [32] that demonstrate their link to genetic variations in clock genes [33]. In this way, and based on genetic predisposition, circadian preferences can be evident in the first moments of life and the genetic influences on these preferences seem to be stable throughout the development of children until adolescence and into later adulthood [30]. Very young children show very marked early riser tendencies; it is estimated that approximately 90% of children under 6 years are early risers and 46% of children between 4 and 11 years are possibly early risers [30]. In adolescents, however, a shift toward the eveningness [30] chronotype is evident. 

Homeostatic and circadian processes control the quality of sleep, and awake periods [34]. The main role of the circadian clock is to promote awakening during the internal biological day and to favor sleep during the internal biological night [34]. However, today, due to unlimited access to artificial light, people exhibit undesirable behaviors regarding their endogenous circadian rhythm [35]. This temporal misalignment, called circadian misalignment, occurs when the internal circadian system is not correctly aligned with the external environment [36] and can lead to chronic sleep deprivation, very common in modern societies [37]. This deprivation can be attributed to quantitative factors, such as insufficient sleep duration, or to qualitative factors, such as fragmented sleep periods [9]. Circadian misalignment is often associated with numerous health problems [35]. One of the effects of circadian misalignment is a reduction in total sleep time, which may also affect sleep architecture since human sleep is composed of rapid eye movement (REM) sleep and non-REM sleep [2,34]. Several factors are responsible for circadian misalignment: the increase in the number of individuals who work in shifts or at night, the increase in the number of working hours, commute times, jet lag, psychosocial stress and engagement with television, radio, and the Internet [9,34,36,38]. Shift work is an essential system in society, being very present in industry as well as in medical institutions [39]. However, shift work is associated with adverse health outcomes, including gastrointestinal disorders, metabolic syndrome, diabetes, reproductive difficulties, and breast and prostate cancer, as well as glucose intolerance and cardiovascular function [40,41]. Caffeine has been shown to have both positive and negative effects on behavior, cognition, and health, depending on the amount consumed. When consumed in excess, it can cause sleep disturbances, since its consumption significantly reduces sleep time and causes disturbances in sleep quality [42]. In recent years, electronic devices, including computers and mobile phones, game consoles and tablets, have been associated with poor sleep among young people [43,44]. Some studies [13,45,46] have linked the mere presence of these devices in the bedroom with the tendency to sleep later, and consequently, shorten sleep duration and increase daytime sleepiness [43,44]. This idea was further reinforced as the use of these devices became the subject of great clinical interest: it was not only shown to increase sedentary behavior but also impoverish the quality of subjective sleep, even leading to falling asleep during school hours and increasing daytime sleepiness [43,44]. In the case of children, among the many factors that affect their sleep time are television viewing and the presence of a television in the bedroom [24]. Television-related behaviors can directly disturb sleep time or increase emotional/mental arousal and light exposure, which can be determinants of sleep duration and quality [24]. These inadequate hours of sleep are associated with poor mental and physical health, which include impaired academic performance, depression, injuries, and an increased risk of obesity [24].

### 3.2. Sleep and Metabolism

All animals, including humans, evolved over millions of years in a stable, seasonal light–dark environment, in which the intervals between light and dark moments could be distinguished within a 24 h period [31,38,47,48,49]. Because of this repetitive and extremely regulated 24 h cycle, an internal circadian clock developed that allows day–night adjustments of metabolic activities, which are at the base of circadian rhythms [44,50]. The internal clock in humans is in the suprachiasmatic nucleus of the anterobasal hypothalamus, functioning as a stopwatch and regulating gene/protein expression, and thus the flow of all the functions of the organism, such as the use and storage of energy, feeding, sleep–wake cycle, electrical activity and concentration of ions and substances, cyclic changes in metabolism and energy homeostasis [31,38,47,48,49]. It is apparent that the basis of the circadian clock is the light–dark cycle. Therefore, the most important external signal is light, which makes individual and physiological behavior correspond to the external day–night cycle, influencing several hormones with metabolic relevance since they present a circadian oscillation with different daily patterns [38,48,50]. Thus, it is believed that changes in the pattern of light–dark exposure or inappropriate exposure to light can affect the circadian rhythm, causing the internal rhythm to become out of sync with the external environment, damaging sleeping behavior and compromising metabolic processes [48]. A metabolic process that is clearly dependent on the light–dark cycle and the function of the suprachiasmatic nucleus is the melatonin cycle [49]. Falling asleep with the television on or sleeping with the light on has been associated with a change in the brain’s natural sleep cycle and melatonin production [51]. The production and secretion of melatonin by the pineal gland occurs in a nocturnal circadian pattern, with the peak being reached 3–5 h after dark, declining precipitously after waking up [49,52,53]). Since melatonin is rapidly released by the pineal gland soon after its production, it can be said that the concentration of melatonin in the blood reflects its synthesis, and fluctuations in melatonin concentration play an important role in the transmission of essential information to the various organs [49]. In addition to its role in transmitting information, melatonin secretion, and the location of its receptors throughout the body, which include the β cells of the pancreatic islets, mean that, according to some authors, melatonin can play an important role in glucose metabolism [54,55]. In fact, performed in vitro tests [54,55] have verified that prolonged exposure of the β islets of the pancreas to melatonin, with the purpose of simulating the period of sleep, increases the sensitivity of the β receptors to glucose [53]. Sleep loss not only affects the melatonin cycle but has also been linked to disturbances in other metabolic functions, specifically an increase in the appetite-stimulating hormone ghrelin [14,16,52,56]. A sleep restriction of 4 h per night for two consecutive days, as well as one night of total sleep restriction, have been shown to increase daytime plasma ghrelin concentrations in young men, mainly in the early hours of the day [6]. However, sleep deprivation also causes a decrease in leptin, the appetite-suppressing hormone, thus favoring food intake by increasing appetite [14,16,52]. Leptin is an amino acid essentially produced by adipocytes, which is secreted in a circadian manner, suggesting that it is influenced by the circadian clock through its sympathetic input in adipocytes [15,57]. However, leptin can also be expressed by non-adipose tissues, such as stomach tissue, and, to this extent, gastric leptin levels show oscillations, being elevated during the night, leading to a reduction in appetite and promoting satiety and night rest, but low during the day, increasing appetite [15,56,57]. Thus, it is suggested that gastric leptin may be involved in appetite regulation by inducing satiety [15,56,57]. Some authors [58] have associated leptin with increased insulin sensitivity as it promotes fat oxidation and reduces fat accumulation in non-adipose tissues. This effect can be directly mediated by leptin due to AMPK activation in certain skeletal muscles and indirectly through the sympathetic hypothalamic axial nervous system [57]. The activation of AMPK, activated protein kinase, leads to the inhibition of acetyl-coenzyme A (CoA) carboxylase [57,59], which leads to a reduction in the intracellular levels of the malonyl CoA metabolite. The entry of fatty acids into the mitochondria decreases and the oxidation of fatty acids is favored [57]. There are published results [60] which indicate that leptin-dependent sleep disturbances may result in an alteration in leptin-sensitive axial hormones. In addition to leptin, adipocytes are also the main producers of adiponectin, which has anti-diabetic, anti-atherogenic and anti-inflammatory properties [57]. Like leptin, adiponectin improves insulin sensitivity thanks to the activation of AMPK and is also responsible for the decrease in hepatic glucose production due to the decrease in mRNA expression of two essential enzymes involved in gluconeogenesis, namely phosphoenolpyruvate carboxylase and glucose-6-phosphatase [57]. Ghrelin, on the other hand, is an anorexigenic peptide produced in the stomach and other organs, such as the pancreas and hypothalamus, but it is mostly released by the stomach and its levels fluctuate based on food intake [15], with a rapid decrease in ghrelin after eating and an increase immediately before a meal [8,15,56]. It has been shown that ghrelin levels increase by 20% before breakfast, 45% before lunch and 51% at dinner [20]. Despite the absence of food intake, it is possible to find high levels of ghrelin during the first hours of the night, with a peak between midnight and two in the morning, which progressively decreases with food intake [15,20,56]. Sleep deprivation causes an increase in circulating ghrelin levels, and this phenomenon is accompanied by an increased feeling of hunger [15]. The way ghrelin stimulates appetite is through the activation of neuropeptide Y, located in the lateral part of the hypothalamus [15]. Ghrelin is responsible for the feeling of appetite and for weight gain and is also responsible for stimulating the release of growth hormones. This hormone is a hypothalamic neuropeptide that regulates eating, energy metabolism, reproduction, and sleep [61]. Regarding the latter, it stimulates the neurons that promote the wake-to-eat cycle, thus modulating arousal and appetite [8]. There are several studies that describe how the growth hormone is controlled through the homeostasis of the wake–sleep cycle, with an increase in its production during sleep being verified in men, namely in stages three and four of slow-wave sleep (SWS). When the sleep period is interrupted, there is a change in the release of growth hormones, the impact being particularly evident in men, but also detectable in women [56]. Another cycle that is influenced by the circadian rhythm is the cortisol profile, which oscillates over the 24 h, since the decrease in its secretion occurs in the early hours of the night and the peak of its secretion occurs at the time of awakening, decreasing progressively throughout the day [50]. Manipulations of the sleep cycle have minimal effects on the cortisol profile, as it is very difficult to detect changes when sleep is interrupted in the morning, coinciding with the peak of corticotropic activity [56]. Sleep deprivation has also been shown to have a negative influence on the response of adrenocorticotropic hormones, adrenaline and on the sensitivity of serotonin (5-HT) receptors, which over time can lead to changes in the system’s response to stress, seen in changes in humor [52]. Serotonin (5′hydroxyptitanin) has been implicated not only in the regulation of emotions, attention and memory, but also in the regulation of appetite and sleep, and its synthesis in the brain is considered critical since for this to occur, the availability of its precursor, the essential amino acid tryptophan, is necessary, and it can only be obtained by humans from the diet [10,62,63].

### 3.3. Sleep and Food Choices

Changes in food choices and eating behaviors are associated with short sleep time [33]. Sleep disturbances have been associated with increased sleepiness and changes in thermoregulatory functions and secretion of the growth hormone by the hypothalamic–pituitary–adrenal gland axis during SWS, which leads to a decrease in energy expenditure [64,65]. This factor was confirmed by Jung et al. (2011), who reported a 7% increase in energy expenditure over 24 h during a day of total elimination of sleep compared to a normal day, which supports the importance of sleep for energy conservation [66], due to a decrease in the practice of physical activity [6,64,65]. However, sleep deprivation also increases the appetite for food intake, and the food choices made at these times result in meals rich in sweet and high-density energy foods, and these phenomena are associated with changes in neuroendocrine control of appetite. Sleep disturbances cause an increase in circulating ghrelin levels and a decrease in leptin levels, which favors an increase in the sensation of appetite and hunger, affecting the energy balance [3,11,28,36,64,65,67,68,69]. The feeling of hunger and appetite due to sleep disturbances makes people choose foods with a high caloric density and rich in carbohydrates, such as sweets, salty snacks, and starchy foods [4,6,33,70,71], verified in girls, with increased intake of sweets and/or fast food and soft drinks in boys [71]. Sleep disorders in children and sleeping less than 7 h/night in adults have been associated with reduced consumption of fruit and vegetables and increased consumption of energy-rich foods with low nutritional value [33]. Adolescents who report sleeping less than 8 h/night tend to consume more total calories from fat than from carbohydrates and protein when compared to those who sleep for 8 or more hours/night [33,36]. In adults, acute sleep deprivation increases caloric intake, mainly due to increased consumption of carbohydrates and fat, as well as increased consumption of snacks [4,70].

### 3.4. Sleep and Chronic Diseases

In modern society, a reduction in sleeping hours is quite common, either for occupational or lifestyle reasons. A short period of sleep, described as sleeping less than six hours a night, sleep deprivation and/or even sleep restriction have been associated with several chronic diseases [72]. Thus, sleep deprivation has been associated with an increased risk of diabetes, obesity, hypertension, breast cancer, coronary heart disease, low bone density, increased body mass index and insulin resistance [3,13,14,38,44,47,73,74,75]. However, excessive sleep duration (more than 9 h/night) is also harmful, being related to an increased incidence of premature mortality, cardiovascular disease, and cognitive damage [47].

### 3.5. Sleep and Stress

Children aged 8–11 years change their energy intake because of changes in their sleep duration [36]. However, sleep deprivation is also responsible for causing physiological stress, which can itself alter energy balance regulators [36]. In a prospective study [76], it was shown that physical and social stress related to family and/or work matters was associated with an increased risk of incidence and persistence of insomnia. Insomnia is characterized by difficulty initiating and/or maintaining sleep, waking up in the early hours of the day and, in general, dissatisfaction with both the quality and quantity of sleep [73].

### 3.6. Sleep and Night Eating Syndrome

Night eating syndrome (NES) is characterized by morning anorexia, hyperphagia in the afternoon and insomnia, and is often due to periods of stress, such as unsuccessful attempts to lose weight [77]. Research demonstrates that patients with NES experience high levels of insomnia and poor sleep quality [78,79,80]. Expanding on this research, when comparing patients with NES with evening hyperphagia to patients with nocturnal ingestions, Loddo et al. found differences in sleep features across NES subgroups. The researchers observed a higher total duration of eating episodes, eating latency following wakening and sleep latency following eating episodes in the evening hyperphagia group [81]. It may be that sleep disturbance is heightened in patients with evening hyperphagia [82]. Zwaan et al. [83] found that across studies, the prevalence of NES in pre-operative bariatric patients ranged from 6 to 64% [83]. The overall prevalence of NES ranges from 2.8 to 8.2% across the eating disorder, obese and bariatric surgery populations [84].

### 3.7. Sleep and Cardiovascular Disease

Currently, there is increasing evidence of a relationship between sleep and the risk of cardiovascular disease [85]. Recently, literature reviews [86,87] have demonstrated the link between dysfunctional sleep patterns and their contribution to the increased risk of cardiovascular disease in shift workers. Evidence supports the existence of a relationship between poor quality and duration of sleep, on the one hand, and the activation of the sympathetic nervous system and increased levels of inflammation, on the other, which are believed to be responsible for inducing endothelial dysfunction, a key factor in the onset of atherosclerosis and consequent increases in the risk of cardiovascular diseases [32,40,85].

### 3.8. Sleep and Insulin Resistance

Scientific evidence [88,89] has shown that acute sleep loss increases food intake, with damage to glucose tolerance and insulin sensitivity, the latter of which increases in the body to maintain glucose homeostasis [75]. Just as important in this whole process is the intestinal hormone glucagon-like peptide 1, GLP-1, secreted after ingesting nutrients by mouth. GLP-1 can improve insulin resistance and reduce food intake; when there is a deterioration of sleep, GLP-1 signaling may be compromised [5]. It was found that in young males, plasma GLP-1 concentrations in the afternoon were reduced after a night of fragmented sleep compared to a regular night of sleep. However, the general concentration of GLP-1 during the 24 h was not affected, which may be due to the subtlety of the intervention, characterized by a small variation in the time spent in REM sleep relative to the time spent in stage two [5]. In other studies [90,91], SWS duration was correlated with insulin sensitivity, although no relationship with arousal was reported. The exact effect that reduced sleep exerts on insulin sensitivity is not yet known, but hormonal mechanisms, particularly changes in hormones responsible for promoting appetite, are increasingly evident [75].

### 3.9. Sleep and Diabetes

The increasing prevalence of type 2 diabetes, as well as the complications that result from it, is a growing concern in terms of public health, both in developed and developing countries [92]. Type 2 diabetes is a disease known to involve a complex combination of genetic factors (such as age and gender) and environmental factors (such as obesity and lifestyle) [29,93,94]. Recently, habitual sleep duration, as well as its quality, have been decreasing because of societal lifestyles [57,75,92,94], and in some epidemiological studies [95,96] individuals who work in shifts have been positively associated with the appearance of type 2 diabetes since this type of work is the main example of a disorder of the circadian rhythm [29]. Scientific evidence has been produced [28,97,98,99] of the existence of a U-shaped relationship (curve morbidity and mortality) between sleep duration and type 2 diabetes, which demonstrates that extreme sleep times in both directions are associated with a higher risk of developing this chronic disease. In a prospective cohort study carried out on middle-aged and elderly people, it was found that those who reported a short sleep duration (5–6 h of sleep/night) were twice as likely to develop diabetes, compared to individuals who had a daily sleep lasting 8 h [57]. At the same time, those who reported a long duration of sleep, longer than 8 h/night, were three times more likely to develop diabetes [57]. Elevated risks remained essentially unchanged even after adjusting for age, hypertension, smoking habits, education, health conditions and waist circumference [57]. Moreover, a 10-year cohort study of 70,026 women, found an increased risk of incident symptomatic diabetes in those who reported a sleep duration of 5 h or less, compared with those who slept between 7 and 8 h [57,100]. On the other hand, in a study carried out in 6898 men and 7392 women, it was shown that leisure activities, moderate/heavy occupation and commuting activities could reduce the risk of type 2 diabetes, as well as decrease total mortality and mortality due to cardiovascular diseases in patients with type 2 diabetes [101].

## 4. Sleep and Sleep Apnea

Excess daytime sleepiness, obesity and sleep apnea are prevalent conditions in developed countries, with obesity being the greatest risk factor for the onset of sleep apnea [102]. Sleep apnea is a common disorder characterized by repetitive partial or total obstruction of the upper airway, leading to episodes of hypoxia, hypoxemia, hypercapnia, and respiratory excitation [9,17]. This disease has a prevalence of 1–3% in healthy children and 2–4% in the general population, but the risk of sleep apnea is increased in obese children and obese adults, in whom its prevalence is estimated to reach 36% and 30%, respectively [17,102]. Sleep apnea thus seems to explain the high level of sleepiness observed in obese individuals. However, despite the association between obesity and sleep apnea, one study showed a weak correlation (r^2^ < 0.3) between the severity of sleep apnea sleep (defined by the apnea–hypopnea index) and the subjective severity of sleepiness (defined by the Epworth Sleepiness Scale) [102]. After using sleep apnea therapy, it would be expected that most people would experience improvements in daytime sleepiness by using continuous positive airway pressure (CPAP) therapy [102]; however, a significant proportion of people with moderate/severe disease continued to experience excessive drowsiness even after treatment [102]. Sleep plays an essential role in maintaining appetite, satiety, and energy balance through hormonal mechanisms such as leptin, an appetite-suppressing hormone, and ghrelin, an appetite-promoting hormone [103]. In turn, sleep apnea increases daytime sleepiness and physical inactivity, and allows for more hours of eating, thus promoting weight gain and obesity. In patients with sleep apnea, high resistance to leptin and high levels of ghrelin, regardless of body mass index, may lead to an increased preference for foods rich in fat, and consequently a greater risk of developing obesity. In this way, the sleep disturbances associated with sleep apnea can cause the promotion of weight gain and may even represent an impediment in weight loss strategies, which would otherwise be effective [103]. A study [104] that investigated the effects of sleep deprivation on food choices in individuals who did not have sleep apnea demonstrated that sleep deprivation increases the desire for high calorie foods and is also responsible for a decrease in activity in the prefrontal and insular regions that regulate appetite and satiety. These results illustrate that hours of insufficient sleep can lead to the development and/or maintenance of obesity through disturbances in brain mechanisms that control appetite.

### Sleep and Obesity

Over the last 3 decades, the prevalence of obesity has increased worldwide, reaching epidemic proportions, and is considered a serious health problem since it is associated not only with type 2 diabetes, but also with cardiovascular diseases, and certain types of cancer [18,27,50]. Some studies have identified several risk factors for the development of obesity, among which low levels of physical activity, sedentary behaviors and easy access to energy-dense foods stand out [18]. The traditional treatment approach consists of restricting the consumption of foods that provide large amounts of energy and increasing physical activity; however, the long-term success of these treatments is relatively low [50]. Parallel to the increase in obesity, in recent decades there has been a reduction in hours of sleep [27,65], suggesting that the short duration of sleep could be a factor favorable to the development of obesity. The main explanation for this phenomenon seems to be simple and consists of the increase in the number of hours available to eat [18,49,65,105,106]. As already mentioned, the endogenous circadian system coordinates daily eating patterns, energy use and storage, alignment of activities, and food consumption, through light from the outside environment. Studies [107] have suggested that circadian rhythm disruption can lead to obesity since, as already mentioned, sleep duration can affect circulating levels of hormones that regulate appetite and caloric intake [16,30], and with them a set of metabolic processes [50]. Several epidemiological data [108,109] support this idea. In the Nurses’ Health Study [108] carried out in 68,183 women, it was found that those who slept 5 h/night had a high body weight when compared to those who had a sleep duration of 7–8 h/night. Multiple cross-sectional studies [110,111,112,113,114] covering a wide age range and both genders, carried out in different countries, were able to identify an association between a chronic period of short sleep and increased body mass index. Several other studies [11,89,115] have shown that an acute but severe restriction of sleep (4 h of sleep) leads to an increased feeling of hunger and energy consumption. Some researchers [11,89,115,116] have also observed an increase in food intake due to the restriction of sleeping hours to 4–5 h/night compared to a sleep period of 8–10 h/night. In cross-sectional studies [19,113] associations were observed between short sleep duration and increased adiposity in children and adolescents. Sleep problems seen in childhood may also contribute to the development of diabetes in adulthood [117]. This conclusion was also presented by another study [118], which reported that the usual duration of sleep is prospectively and independently associated with obesity and mortality, and children with an inadequate number of hours of sleep are those who are more likely to become obese.

## 5. Discussion/Conclusions

The evidence found in carrying out this bibliographical review allowed us to verify that since ancient times human beings have carried out their daily tasks during the day, and rested and recovered during the night, which allows them to replenish spent energy and prepare for tasks to be performed on the following day. Due to the light–dark cycle, which remained unaltered for several million years, humans developed an endogenous circadian system that remained in concordance with the external circadian rhythm and allowed the human organism to maintain adequate homeostasis for the endogenous circadian rhythm, responsible, in turn, for regulating many of the vital systems for the proper functioning of the human body, such as the immune, nervous, and metabolic systems. Currently, changes in sleep patterns are increasingly evident, and this fact is mainly due to working conditions as well as lifestyles. However, there is another important aspect that impairs the period of sleep, which is the growing dependence on electronic devices, such as computers, tablets, cell phones, radios, and artificial light. All these factors provide conditions that disrupt the proper circadian rhythm, consequently leading to disturbances in the various systems dependent on it. A disruption of the various systems controlled by the internal circadian clock, such as the metabolic system, responsible for controlling hormones such as ghrelin and leptin, related to the feelings of appetite and satiety, respectively, can lead to a change in the pattern of food consumption, giving preference to foods with greater caloric density in an attempt to compensate for the feeling of hunger, as well as feeling more tired due to being awake during a period of the day intended for rest and restoration of energy. Inappropriate choices can lead to the development of several chronic diseases that are very common in modern society, such as diabetes, cardiovascular disease, and obesity. 

A total of 112 articles published in PubMed were used to carry out the literature review, as the most credible database with more than 35 million citations and abstracts of biomedical studies, but the research could also have been complemented with other databases, such as Scopus. The quantity of literature derived from the first searches was huge, so a refinement of the keywords used was required in the search process to obtain only articles that were focused on “Sleep Patterns”, “Eating Behavior” and “chronic noncommunicable diseases”.

Regarding a possible bias related to the authors’ personal viewpoints, they were careful to not express any opinion throughout the text, only discussing and drawing conclusions based on the scientific evidence obtained in the analyzed articles.

By including articles on this topic over a period of 22 years, the probability of bias associated with gaps in literature search practices that may lead to the omission of relevant research is low. However, there may be some bias related to the translation of data from the primary literature to summarization in the review, misrepresentation, or misinterpretation of original source data.

To our knowledge, this is the first review of research addressing the relationship between sleep patterns, on the one hand, and eating behaviors and the risk of noncommunicable diseases, on the other. The review offers a valuable overview of the research literature in this field for an extended period of more than 20 years.

It can therefore be concluded that sleep, as well as the period allocated to it, is extremely important for the optimal functioning of the most diverse functions of the organism, and that today it is considered of secondary importance, being therefore impaired, leading to changes in the functions performed by the various body systems. These alterations cause sleep-deprived people to make inappropriate food choices, thus favoring the onset of chronic diseases.

## Data Availability

Not applicable.
